# Extracellular Vesicles in Renal Pathophysiology

**DOI:** 10.3389/fmolb.2017.00037

**Published:** 2017-06-07

**Authors:** Margherita A. C. Pomatto, Chiara Gai, Benedetta Bussolati, Giovanni Camussi

**Affiliations:** ^1^Stem Cell Laboratory, Department of Medical Sciences, University of TurinTurin, Italy; ^2^Department of Molecular Biotechnology and Health Sciences, University of TurinTurin, Italy

**Keywords:** extracellular vesicles, intercellular communication, kidney, physiology, pathology, biomolecules, biomarkers

## Abstract

Extracellular vesicles are a heterogeneous population of microparticles released by virtually all living cells which have been recently widely investigated in different biological fields. They are typically composed of two primary types (exosomes and microvesicles) and are recently commanding increasing attention as mediators of cellular signaling. Indeed, these vesicles can affect recipient cells by carrying and delivering complex cargos of biomolecules (including proteins, lipids and nucleic acids), protected from enzymatic degradation in the environment. Their importance has been demonstrated in the pathophysiology of several organs, in particular in kidney, where different cell types secrete extracellular vesicles that mediate their communication with downstream urinary tract cells. Over the past few years, evidence has been shown that vesicles participate in kidney development and normal physiology. Moreover, EVs are widely demonstrated to be implicated in cellular signaling during renal regenerative and pathological processes. Although many EV mechanisms are still poorly understood, in particular in kidney, the discovery of their role could help to shed light on renal biological processes which are so far elusive. Lastly, extracellular vesicles secreted by renal cells gather in urine, thus becoming a great resource for disease or recovery markers and a promising non-invasive diagnostic instrument for renal disease. In the present review, we discuss the most recent findings on the role of extracellular vesicles in renal physiopathology and their potential implication in diagnosis and therapy.

## Introduction

Cell-to-cell communication is a very complex and finely regulated system which ensures proper signaling among different cell types in tissues. Aside from soluble factors, cell-derived extracellular vesicles (EVs) were described as a new mechanism of communication between cells (Ratajczak et al., 2006; Cocucci et al., 2009). It has been widely proven that EVs confer stability to enclosed proteins and nucleic acids, by protecting them from enzymatic degradation, and mediate the entry into specific recipient cell types (Bobrie et al., 2011; Chaput and Théry, 2011; Lee et al., 2012; Ratajczak et al., 2012; Hoshino et al., 2015). Since their discovery roughly 30 years ago (Trams et al., 1981; Pan and Johnstone, 1983; Harding et al., 1984), EVs were purified not only from nearly all mammalian cell types and body fluids, but also from lower eukaryotes, prokaryotes, and plants (Yáñez-Mó et al., 2015). This suggests that EV-mediated cell signaling emerged very early during evolution as a primitive and essential mechanism of cell communication (Ratajczak et al., 2006).

Importantly, EVs have been involved in the pathophysiology of different organs. In pathological conditions, for example, EVs take part in tumorigenesis. They may modulate cell-to-cell communication within the tumor microenvironment, play a role in drug resistance and, migrating from the tumor to distant niches, promote metastasis formation (Wendler et al., 2017). EVs were also shown to carry and spread through the brain misfolded proteins involved in several neurodegenerative diseases, such as prion, Alzheimer's, and Parkinson's disease, and amyotrophic lateral sclerosis (Quek and Hill, 2017). Evidence showed EVs' involvement in pathophysiology of several liver diseases (Maji et al., 2017) and autoimmune diseases like Systemic Lupus Erythematosus and Multiple Sclerosis (Ulivieri and Baldari, 2017).

On the other hand, EVs participate in many physiological processes, such as adaptive and innate immunity (Maas et al., 2017), stem cell maintenance (Ratajczak et al., 2006), bone calcification, central processes of embryogenesis, liver homeostasis (Yáñez-Mó et al., 2015), and the coagulation cascade (Del Conde et al., 2005). EVs are involved in reproductive processes, such as gametogenesis, fertilization, and implantation of the embryo (Machtinger et al., 2016). Regarding their role in organs, it was demonstrated that EVs mediate the communication between neurons in the brain, contributing to local and distal synaptic plasticity (Budnik et al., 2016). In the kidney, EVs were recognized to participate in the pathophysiology by mediating intercellular communication, transferring their content, activating signaling pathways in target cells, or just representing a route of disposal for cellular contents (Hogan et al., 2009; Borges et al., 2013). Although the presence of urinary EVs (uEVs) was first reported in the early 1990s (Sato et al., 1990), these vesicles were fully characterized only in 2004 (Pisitkun et al., 2004). Recently, it has been shown that uEVs are actively released by almost all renal cells along the nephron and the urogenital tract as well as by infiltrating inflammatory cells (Ranghino et al., 2015). EVs can be uptaken along the urogenital tract and can affect the function of recipient cells (Knepper and Pisitkun, 2007). Initially, it was thought that their main physiological role is the excretion of cell debris, such as proteins and lipids (van Balkom et al., 2011). However, the significant amount of energy probably required for EVs excretion and their impact in other different physiological functions (Knepper and Pisitkun, 2007) suggested EVs as potential mediators of intra-renal signaling. Moreover, variations in number, origin or content of EVs isolated from urine may signal an alteration in the physiopathological state of the kidneys (Ranghino et al., 2015). For this reason, uEVs raised a great interest as a putative useful tool for non-invasive diagnosis. Furthermore, EVs derived from stem cells showed regenerative properties that may be applicable in renal pathologies. For instance, it has been demonstrated that mesenchymal stem cells (MSCs) have a renal regenerative activity and MSC-derived EVs have been implicated as the main paracrine players (Bruno et al., 2016).

In this review, we will give an overview of EV features and we will discuss their role in renal physiology and disease. Moreover, we will describe the potential of uEVs as biomarkers of renal diseases.

## EV biogenesis and composition

EVs represent a mixed population of microparticles commonly categorized on their biogenesis, size and surface markers. The main classes of non-apoptotic EVs are exosomes and microvesicles (El Andaloussi et al., 2013; Katsuda et al., 2013; Helmke and von Vietinghoff, 2016). Exosomes are derived from the endosomal compartment and show a variable size ranging approximately from 40 to 150 nm (Greening et al., 2015). They are stored within multivesicular bodies (MVBs) of the late endosome that fuse with the cell membrane and release their content (Théry et al., 2009; Mathivanan et al., 2010; György et al., 2011). The exact mechanism of exosome assembly and sorting is not completely elucidated. However, different mechanisms of exosome biogenesis have recently been identified (Raiborg and Stenmark, 2009; Bobrie et al., 2011; Baietti et al., 2012; Nabhan et al., 2012). The endosomal sorting complex required for transport (ESCRT)-III forms spirals that induce the inward budding and fission of vesicles to form MVBs (Chiaruttini et al., 2015; Lee et al., 2015; McCullough et al., 2015). The ESCRT machinery also seems to be recruited by viruses for budding at the plasma membrane of host cells and release (Gan and Gould, 2011; Lindenbach, 2013). Small GTPases (such as RAB11, RAB27A, and RAB31) are implicated in the fusion of MVBs with the cell membrane (Ostrowski et al., 2010; Bobrie et al., 2011). Cytoskeleton activation, under the regulation of p53 protein, was showed to regulate the exocytosis of exosomes (Yu et al., 2006). Moreover, ceramide formation is important in exosome biogenesis (Trajkovic et al., 2008), and ceramide synthesis modifies exosomes cargo (Gatti et al., 2011; Maas et al., 2017).

Microvesicles, also known as shedding vesicles, are larger than exosomes and represent a more heterogeneous population of vesicles originated by budding of cell surface (Théry et al., 2009; El Andaloussi et al., 2013; Greening et al., 2015). This process is regulated by membrane lipid microdomains and the dynamic contraction of the plasma membrane, which is controlled by proteins such as ADP-ribosylation factor 6 (ARF6) (Muralidharan-Chari et al., 2009; De Palma et al., 2016a). Moreover, levels of calcium impact on specific enzymes, including flippase, floppase, and scramblase which modify the asymmetry of plasmamembrane phospholipids (Hugel et al., 2005). Increased calcium levels promote the transfer of phosphatydilserine (PS) toward the inner membrane by inhibiting flippase. This process is ATP-dependent (Dignat-George and Boulanger, 2011) and activates scramblase leading the shift of PS from the inner to the outer leaflet of the cell membrane (Abid Hussein et al., 2005). The activation of cytosolic proteases, such as gelsolin and calpain, by calcium, promotes the detachment of plasma-membrane protrusions from the cortical actin (Cocucci et al., 2009).

Since microvesicles are secreted in conjunction with exosomes and share several of their functions and characteristics, it has been proposed to collectively identify them as EVs (Gould and Raposo, 2013). EV cargo is various and includes cytoplasmic proteins, surface receptors, certain lipid raft-interacting proteins, DNAs, and RNAs (Théry et al., 2009; Lee et al., 2012). In general, lipids enriched in EV membranes are glycosphingolipids, sphingomyelin, cholesterol, and phosphatidyl-serine (Llorente, 2013; Bruno et al., 2016). EVs are constitutively released by cells and their secretion is regulated by the cellular machinery. Exosome production, for instance, depends on the cell-specific expression and activity of Rab GTPases and on the interaction between MVBs and microtubule network (Villarroya-Beltri et al., 2014). Furthermore, EV secretion is known to be increased in cellular stress conditions including hypoxia, starvation, changes in pH membrane, shear stress, oxidative stress, thermal changes, irradiation, inflammation (King et al., 2012; Buzas et al., 2014; Roma-Rodrigues et al., 2014) and several stimuli, such as the activation of a signaling cascade or the membrane depolarization (Raposo et al., 2013).

## EV uptake and cargo

**Figure 1:**
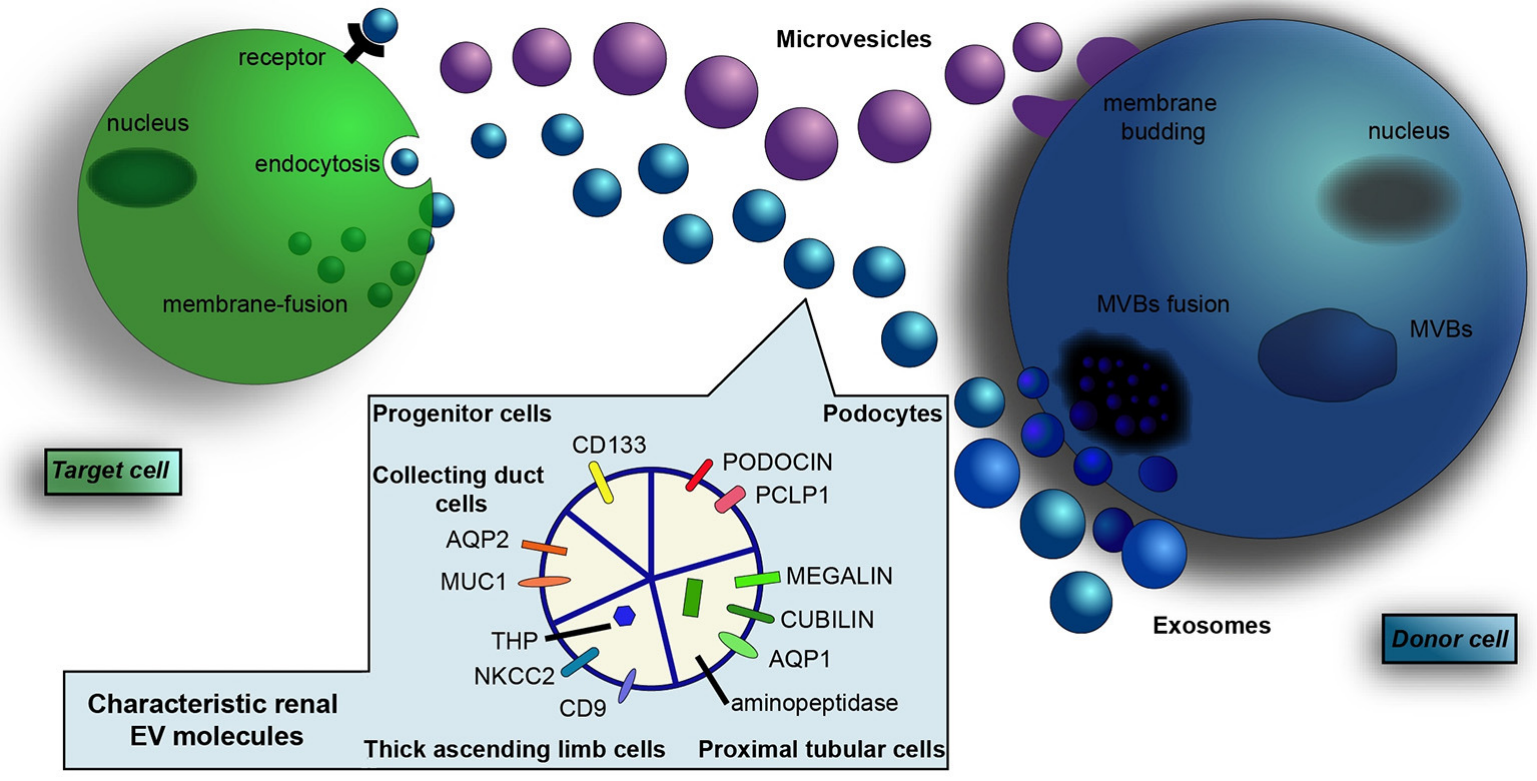
Renal-derived extracellular vesicles. Extracellular vesicles (EVs) are a heterogeneous population of microparticles, mainly composed by exosomes and microvesicles. In particular, exosomes (in blue) are stored within multivesicular bodies (MVBs) of the late endosome and are released in the microenvironment after fusion with the cell membrane, whereas microvesicles (in violet) originate by direct budding from the cell surface. After their secretion, EVs exert their effects on adjacent or distant recipient cells in a pleiotropic manner, directly activating cell surface receptors, blending with cell membrane or by endocytic uptake and transferring their cargo inside cells. EVs contain a complex cargo of biomolecules that include proteins, surface receptors, lipids, transcription factors, genes, mRNAs, and miRNAs. Their content mirrors the cell of origin and EVs collected from urine contain proteins and transporters specific of renal and urogenital tract epithelial cells. In particular, the presence of podocin and podocalyxin (PCLP1) is characteristic of glomerular podocytes, whereas the expression of megalin, cubilin, aminopeptidase and aquaporin-1 (AQP1) indicate proximal tubular cell source. Moreover, EVs from the thick ascending limb of the Henle's loop contain Tamm Horsfall protein (THP), CD9, and type 2 Na-K-2Cl cotransporter (NKCC2). EVs from the collecting duct carry aquaporin-2 (AQP2) and mucin-1 (MUC1), whereas the expression of CD133 marker identify renal progenitor cells.

EVs were recognized as important vectors of information acting in a paracrine manner to regulate gene expression and modulate the phenotypes of adjacent or distant recipient cells (Turturici et al., 2014). It was observed that EVs are internalized within target cells in several ways (Figure 1). First, they can enter through surface ligands interaction, thus in specifically recognized target cells (Lösche et al., 2004; Turturici et al., 2014). For example, dendritic cell-derived EVs carry intercellular adhesion molecule 1 that specifically binds lymphocyte function-associated antigen 1, which is exposed on activated, but not resting, T cells (Nolte-'t Hoen et al., 2009). EVs derived from proangiogenic progenitors present on their membrane α4 and β1 integrins and L-selectin, which interact with and mediate the uptake by recipient endothelial cells (Deregibus et al., 2007). Then, EVs can be internalized by clathrin-dependent endocytic mechanisms, as micropinocytosis, caveolin-mediated internalization, phagocytosis, and lipid raft–mediated uptake (Mulcahy et al., 2014). Recently, evidence has highlighted that vesicle composition and microenvironmental conditions may influence EV uptake. It was demonstrated that a high lipid raft content in EVs facilitates their fusion with cells (Mulcahy et al., 2014; Maas et al., 2017) and lipid rafts are involved in EV uptake in the kidney (Gildea et al., 2014). In addition, acidic pH in the extracellular environment enhances EV–membrane fusion (Parolini et al., 2009; Maas et al., 2017), and, in tumors the acidic microenvironment promotes the release of EVs with higher cell fusion capacity (Parolini et al., 2009). However, it is still unclear if different interaction mechanisms coexist in the same cell or vary depending on recipient cell type and EV origin. A recent work has shown how the uptake of T lymphocyte-derived EVs by human retinal endothelial cells is regulated by either temperature, extracellular calcium, and the expression levels of the low-density lipoprotein receptor (LDLR) (Yang et al., 2012).

A peculiar aspect of EVs is the ability to protect their cargo against degradation and facilitate its intracellular uptake (Arroyo et al., 2011; Lai et al., 2015). Indeed, EVs can transfer functional proteins, bioactive lipids, transcription factors, genes, mRNAs, and miRNAs (Ratajczak et al., 2006; Deregibus et al., 2007; Valadi et al., 2007; Skog et al., 2008; Yuan et al., 2009). Many reports showed that proteins and miRNA cargo are selectively sorted into EVs (Deregibus et al., 2007; Valadi et al., 2007; Collino et al., 2010; Bolukbasi et al., 2012; Montecalvo et al., 2012; Koppers-Lalic et al., 2014; Cha et al., 2015). The endosomal sorting complex required for transport (ESCRT) associated with programmed cell death 6 interacting protein (PDCD6IP; also known as Alix) and tumor susceptibility gene 101 protein (TSG101) regulates cargo sorting into exosomes (Raiborg and Stenmark, 2009; Baietti et al., 2012; Nabhan et al., 2012). A recent work has shown that Alix, a multifunctional protein of the ESCRT complex, interacts with argonaute protein-2 (Ago2), which is involved in miRNA biogenesis, and the complex participates in driving miRNAs within EVs (Iavello et al., 2016).

In particular, the miRNA cargo plays a key role in EVs biologic activity and modulate protein levels of targeted genes (Gracia et al., 2017). It was shown that tumor-suppressive miRNAs carried by stem cell-derived EVs inhibit tumor growth (Fonsato et al., 2012; Bruno et al., 2013, 2014). Moreover, EVs from MSCs showed to induce a recovery after acute kidney injury (AKI) *in vivo* through the transfer of miRNAs (Collino et al., 2015). EVs from urinary tract include renal-derived EVs and showed to carry mostly ribosomal and non-coding RNAs, such as miRNAs, but also small amount of DNA and mRNAs for proteins specific to the nephron and all the genitourinary system (Miranda et al., 2010; Ranghino et al., 2015). Of note, these urinary EVs show a RNA profile comparable to that of kidney tissue, including the presence of 18S and 28S rRNA, which is normally scarcely present in cell line-derived EVs (Dear, 2014).

## EVs in renal physiology

The kidney is a vital organ that, among its many functions, ensures the filtration of the blood. The glomerular filtration apparatus prevents EVs contained into the blood to enter the lumen of renal nephron (Pisitkun et al., 2004). Thus, it is plausible that EVs secreted into extracellular fluids have roles in renal signaling solely by stimulating cell types that face the vascular compartment and cells of the immune system (van Balkom et al., 2011). It is therefore possible that intra-nephron EVs, exclusively originated from the urinary tract, may have a role in renal processes (Pisitkun et al., 2004). A few years ago, it was shown for the first time that EVs are involved in intra-renal signaling by demonstrating that exosomes from collecting duct cells can induce the expression of aquaporin 2 (AQP2) in recipient cells (Street et al., 2011).

The content of EVs conveyed into urine (uEVs) reflects their cells of origin, with specific proteins (Dimov et al., 2009), mRNAs (Miranda et al., 2010), and miRNAs (Alvarez et al., 2012) and painstakingly mirrors the expression levels of donor cells (Miranda et al., 2010). In fact, it was shown that a selective knockout of a collecting duct-selective marker (V-VATPase-B1) in mice deleted this marker from urinary EVs (Miranda et al., 2010). Moreover, uEVs showed to contain proteins and transporters specific of renal and urogenital tract epithelial cells (Figure 1). For example, EVs from glomerular podocytes express podocin and podocalyxin (Hogan et al., 2014); EVs from proximal tubular cells contain megalin, cubilin, aminopeptidase (Moon et al., 2011), and aquaporin-1 (AQP)-1; EVs from the thick ascending limb of the Henle's loop carry CD9, type 2 Na-K-2Cl cotransporter (NKCC2), and Tamm Horsfall protein (THP) (Ranghino et al., 2015); EVs from collecting ducts carry AQP-2 and mucin-1 (Pisitkun et al., 2004; Gonzales et al., 2009). Moreover, CD133 was recognized as a marker of renal progenitor cells (Dimuccio et al., 2014).

**Figure 2:**
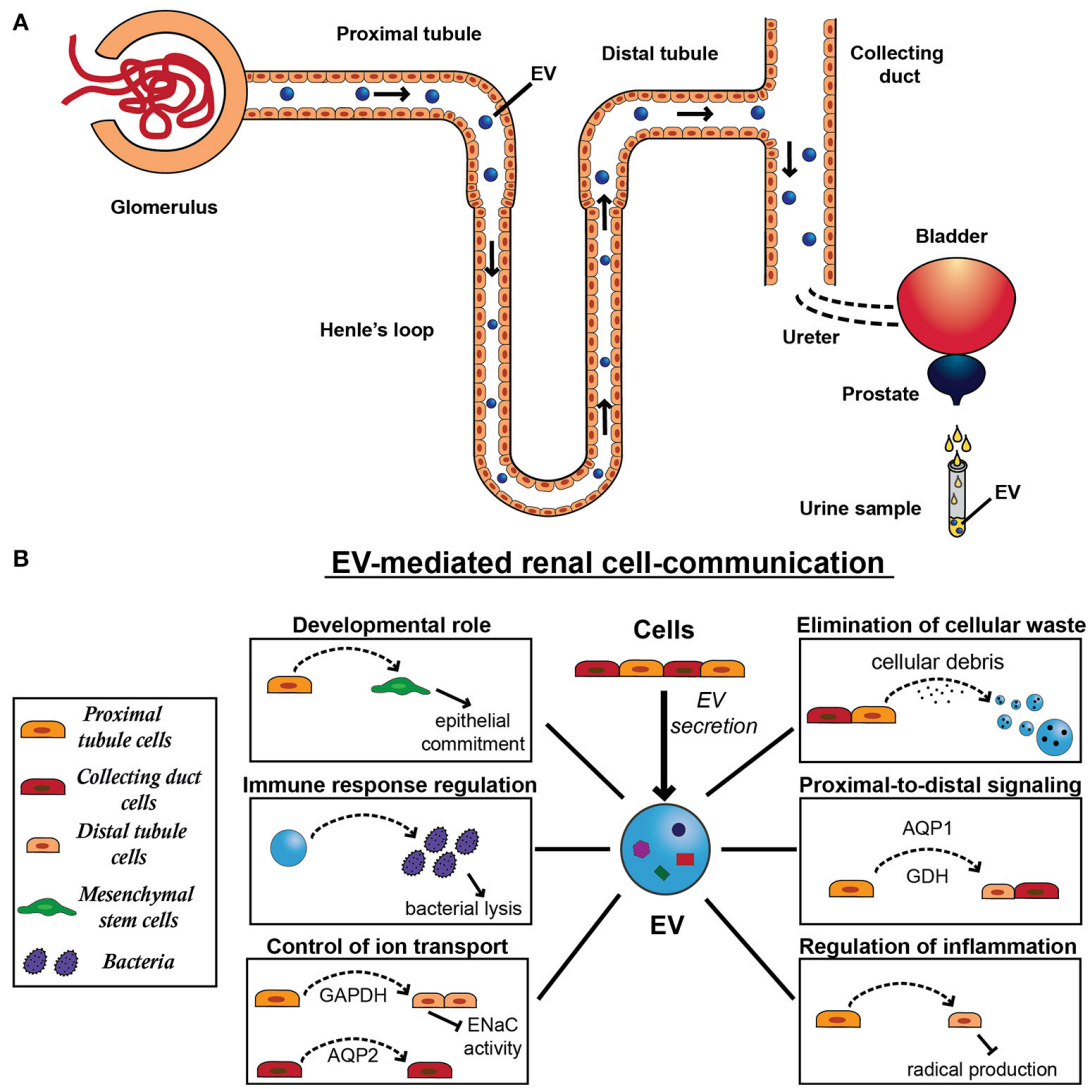
Extracellular vesicle secretion and physiological function in the kidney. **(A)** All cell types of the nephron that face the urinary space secrete EVs, starting from the glomerular podocytes through the proximal tubule, the limb of Henle, the distal tubule, and the collecting duct. After their secretion, EVs can be uptaken by downstream cells, influencing recipient cell behavior. Alternatively to their action on cells, EVs can cross the urinary tract and pass through following organs, including ureters, bladder, prostate, and urethra. EVs released by resident epithelial cells congregate with renal EVs and ultimately conveyed in the urine, providing a source of physiopathological markers of the urinary tract. **(B)** EV-mediated renal communication seems to be a physiological system of cell signaling and involves several EV roles, including elimination of cellular waste, proximal-to-distal signaling, developmental roles, control of ion transport, regulation of inflammation and immune response. In fact, EVs released by proximal tubule cells can be uptaken by distal tubule and collecting duct cells transferring tubular proteins, such as aquaporin-1 (AQP1) and the ammonium-generating enzyme glutaminase (GDH). EVs can also mediate the transfer of another aquaporin member, aquaporin-2 (AQP2) between cortical collecting duct cells. Moreover, by carrying active GAPDH, proximal tubule cells can regulate the renal transport of sodium through EVs, decreasing ENaC activity in distal tubule and collecting duct cells. Similarly, these EVs can also transfer anti-inflammatory message from proximal tubular cells exposed to dopamine receptor agonist and induce a decrease in cell radical production in distal tubular cells. Moreover, EVs derived from tubular cells are implicated in an important process for nephrogenesis and mediate the induction of the mesenchymal-to-epithelial transition (MET) in mesenchymal stem cells (MSCs). Finally, urinary EVs can induce bacterial lysis, contributing to the immune response in the urinary tract.

Despite the role of renal EVs is not yet completely understood up today, recent findings demonstrated their importance in several mechanisms, as discussed below (Figure 2).

### Elimination of cellular waste

After their secretion, EVs can be eliminated as cellular waste. This might be a more efficient strategy for the elimination of senescent proteins compared to proteasomal and lysosomal degradation (van Balkom et al., 2011).

### Proximal-to-distal signaling

On the other hand, EVs can be uptaken downstream, affecting the function of recipient cells (Dimov et al., 2009; Figure 2A). Notably, uEVs abundantly express CD24, a small glycosylphosphatidylinositol-anchored molecule expressed both by tubule cells and podocytes (Keller et al., 2007), which is involved in cell-cell adhesion and signaling (Dimov et al., 2009). Moreover, uEVs seems to specifically interact with recipient cells through primary cilia (Hogan et al., 2009). This observation is supported by data from a biliary model demonstrating that exosome signaling affects ERK signaling, miRNA expression, and cell proliferation (Masyuk et al., 2010). Moreover, molecules present in urine can influence EV uptake into recipient cells. It has been conjectured that, in downstream nephron segments, the EV fusion with cells could be limited by THP, an abundant polymeric protein in normal urine (van Balkom et al., 2011). Indeed, EVs may provide a way for proximal-to-distal signaling, and, for example EVs from podocytes can pass through the renal tubule and transmit information to epithelial cells of the collecting duct (Prunotto et al., 2013; Salih et al., 2014). It was demonstrated that both distal tubule and collecting duct cells can take up and store into MVBs the EVs released by proximal tubule cells (Gildea et al., 2014). This may explain why proteins typically expressed by tubule cells have been detected in downstream nephron segments, including the water channel aquaporin-1 (AQP1) (Sabolic et al., 1992) and the ammonium-generating enzyme glutaminase (Figure 2B; Wright et al., 1990, 1992).

### Regulation of inflammation

Moreover, it was observed that EVs from proximal tubular cells cultured in presence of a dopamine receptor agonist can decrease radical production in distal tubular cells, indicating the transfer of an anti-inflammatory message (Figure 2B; Gildea et al., 2014; Bruno et al., 2016).

### Control of ion transport

EVs can also mediate the communication between proximal and distal tubules and collecting ducts to regulate the transport of sodium. In fact, Jella et al. demonstrated that EVs from proximal tubule cells carry active GAPDH that decreases ENaC activity by reducing the channel's open probability in distal tubules and collecting ducts (Figure 2B; Jella et al., 2016). EVs can also mediate the transfer of aquaporin-2 (AQP2) between cortical collecting duct cells, increasing both AQP2 expression and water transport in recipient cells (Figure 2B; Street et al., 2011). In this study, desmopressin was used to stimulate an increase in AQP2 content and Oosthuyzen et al. have recently demonstrated that this vasopressin analogue selectively stimulated EV uptake in tubular cells, whereas a vasopressin antagonist reduced the uptake of injected EVs within renal tissue *in vivo*. This suggests that uEVs signaling is a physiologically regulated process (Oosthuyzen et al., 2016). Moreover, they demonstrated that this mechanism can be used to deliver miRNAs to collecting duct cells resulting in downregulation of target transcripts (Oosthuyzen et al., 2016). Furthermore, uEVs showed to be enriched with angiotensin-converting enzyme (Pisitkun et al., 2004; Gonzales et al., 2009), which may have a role in the renin-angiotensin system hence playing a part in water balance (Navar et al., 2002).

### Regulation of immune response

A novel role of uEVs has recently emerged: the stream of EVs from renal tubular epithelia can contribute to the immune response in the urinary tract. Thus, the anatomic structure of the urinary system results in its continuous exposure to bacterial infections contrasted by a highly effective innate immune response. It has been shown that uEVs are highly enriched in innate immune proteins and inhibit the growth of the most common human urinary pathogen *E. coli* through bacterial lysis (Hiemstra et al., 2014), highlighting their role in the immune mechanism in the urinary system. This finding is consistent with a previously report of protection against influenza A virus infection by EVs derived from respiratory epithelium in canine kidney cells *in vitro* (Kesimer et al., 2009). Moreover, uEVs expressing tissue factor (TF) could supply additional sources of TF that could support hemostasis and coagulation. Thus, uEVs could reduce blood loss and the hazard of microorganisms entering the body through urinary and urethral epithelia, contributing to host defenses (Kleinjan et al., 2012). An interesting work demonstrated that uEVs can synthesize ATP aerobically by consuming oxygen (Bruschi et al., 2016).

### Developmental roles

Eventually, EVs can play a key role in kidney development and regeneration mediating the interaction between epithelial cells and mesenchymal cells (Figure 2B; Chiabotto et al., 2016). In fact, tubular epithelial cells (TECs) can induce an epithelial commitment in MSCs through activation of mesenchymal-to-epithelial transition, an essential process for nephrogenesis and kidney embryonic development, which allows the formation of a tubular epithelial structure from the metanephric mesenchyme (Singaravelu and Padanilam, 2009; Kanazawa et al., 2011). Recently, Chiabotto et al. (2016) demonstrated that this epithelial differentiation is mainly mediated by EVs derived from TECs and, in particular, by a small subassembly of miRNAs belonging to the miR-200 family able to induce a long-term modification in MSC transcriptome.

## EVs in kidney diseases

EVs' role in renal communication is not only involved in physiological processes, but also in pathological conditions. Indeed, the transfer of information mediated by EVs may participate to the biological mechanisms that lead to diseases or, in contrast, be mediator of benignant pathways essential for disease recovery.

### Cancer

Tumors are surrounded by a complex microenvironment and cancer cells require an active exchange of information with neighboring cells, including endothelial cells, fibroblasts, pericytes, and infiltrating immune cells (Hanahan and Weinberg, 2011). In this context, several cell types communicate to promote or suppress disease progression (Kohlhapp et al., 2015) and EVs were shown to take part in these signalings (Zomer and van Rheenen, 2016). In fact, it is generally accepted that tumor cells release EVs capable of organizing tumor progression stimulating survival essential processes, such as proliferation, angiogenesis, metastasis formation, and immune-escape (Lopatina et al., 2016). A fundamental stage to ensure tumor development is the inhibition of immune surveillance. Tumor-derived EVs (tEVs) were reported to induce tolerance by different mechanisms. For example, our group have recently demonstrated that EVs derived from renal cancer stem cells impaired monocyte differentiation and maturation into dendritic cells by strongly reducing the expression of HLA-DR, costimulatory molecules, and adhesion molecules (Grange et al., 2015). This immune-modulatory role was correlated to the presence of HLA-G, which is known to inhibit immune response thus favoring cancer immune escape. Moreover, Wieckowski et al. (2009) demonstrated that tEVs can directly promote T regulatory cell expansion and the demise of antitumor CD8+ effector T cells. tEVs may also induce apoptosis in lymphocytes by carrying ligands for death receptors TRAIL and FasL (Andreola et al., 2002; Huber et al., 2005). Furthermore, tEVs can regulate monocyte differentiation, endorsing the myeloid immunosuppressive phenotype of these cells (Valenti et al., 2006) and an immunosuppressive macrophage phenotype (de Vrij et al., 2015).

Beyond their effect on immune cells, cancer EVs may alter the functions of non-immune cells within the tumor stroma and they are involved in the differentiation of fibroblasts into myofibroblasts, which secrete ECM components and can support tumor progression (Hanahan and Weinberg, 2011). In fact, Sidhu et al. (2004) demonstrated that EVs derived from lung carcinoma cells carry EMMPRIN to fibroblasts inducing the production of matrix metalloproteinases (MMPs) and enabling tumor invasion and metastasis. Recently, Webber et al. (2010) showed that tEVs express TGF-β1 protein on their outer surface and trigger myofibroblast activation. During tumor growth, hypoxia promotes survival and propagation of tumor cells by influencing the stroma (Finger and Giaccia, 2010) and inducing the release of neovascularization-stimulating factors (Maas et al., 2017). It was observed that the hypoxic microenvironment can affect tEVs composition (Becker et al., 2016) and enhance their angiogenetic and metastatic potential (Park et al., 2010). These alterations can also include the miRNA content. It was shown that hypoxia enhances the compartmentalization of pro-angiogenic miRNAs such as the miR-210 in tEVs (King et al., 2012), or miR-126 and miR-296 in EVs derived from proangiogenic progenitors (Cantaluppi et al., 2012).

In addition, altered vascularization promoted by cancer cells might be dependent on the secretion not only of known angiogenic cytokines and growth factors, but also of EVs (Janowska-Wieczorek et al., 2005; Iero et al., 2008; Skog et al., 2008). In fact, our group found that EVs released by renal cancer stem cells specifically display proangiogenic properties able to favor tumor vascularization (Grange et al., 2011). These vesicles were enriched with proangiogenic mRNAs, miRNAs, and proteins (MMP2/9, angiopoietin 1, ephrin A3, FGF, and VEGF) that could favor tumor vascularization and lung metastasis by priming endothelial cells. Moreover, miRNAs enriched in tEVs included miRNAs described as significantly up-regulated in patients with ovarian, colorectal, and prostate cancer (PCa) (miR-200c, -92, and -141), associated with tumor invasion and metastases (miR-29a, -650, and -151), and directly associated with RCC (miR-19b, -29c, and -151) (Grange et al., 2011). tEVs can also stimulate endothelial cell migration and *in vivo* angiogenesis via their membrane sphingomyelin (Kim et al., 2002). Moreover, tEVs are enriched in MMPs (Taraboletti et al., 2002) as well as in the extracellular MMP inducer CD147 (Millimaggi et al., 2007), promoting the degradation of extracellular matrix proteins necessary for the angiogenic process. tEVs also contain and deliver, at both mRNAs and protein level, pro-angiogenic modulators such as vascular endothelial growth factor (VEGF), fibroblast growth factor (FGF), epidermal growth factor receptor (EGF-R), interleukin 6 (IL-6), interleukin 8 (IL-8), angiogenin, and anti-angiogenic proteins like TIMP-1 and -2 (Skog et al., 2008). Via their cargo, tEVs can alter the fate of normal cells. In particular, renal cancer stem cell-derived EVs can induce a pro-tumorigenic phenotype in recipient MSCs increasing the expression of genes associated with matrix remodeling (COL4A3), cell migration (CXCR4, CXCR7), tumor growth (IL-8, Osteopontin and Myeloperoxidase) and angiogenesis. Importantly, EV-stimulated MSCs showed an enhanced capacity to induce migration of renal tumor cells and vessel-like formation *in vitro* and supported tumor development and vascularization *in vivo* (Lindoso et al., 2015). This finding is consistent with other studies reporting that EVs can favor local and distant spread of tumor cells, promoting tumor migration and invasion. For example, it was observed that tEVs promote the recruitment of different cell types (such as fibroblasts, endothelial cells, macrophages, and various populations of bone marrow-derived cells) to the pre-metastatic niche (Peinado et al., 2012; Costa-Silva et al., 2015) and may be important priming factors that help to establish metastatic niches, typically by interacting with normal host cells (Maas et al., 2017). The comparison of EVs from PCa stem cells with that from the bulk tumor showed a selectively pattern of miRNAs that may contribute to local invasion and pre-metastatic niche formation through fibroblast migration (Sanchez et al., 2016). In fact, highly abundant exosomal miRNAs, such as miR-100-5p, miR-21-5p, and miR-139-5p, increased MMPs 2, 9, and 13, RANKL expression, and fibroblast migration upon transfection into prostate fibroblasts (Sanchez et al., 2016).

### Non-cancer diseases

Besides their role in tumors, EVs also gained attention as mediators of several other pathological conditions, such as endothelial dysfunctions, immune system alterations, fibrosis, and inflammation (Prado et al., 2008). Thus, EVs have a role in modulation of the microenvironment and in amplification of kidney damage or recovery, as shown by the involvement of blood-derived EVs in hypertension, graft rejection, and in several glomerulopathies (van Balkom et al., 2011).

#### EVs and endothelium

EVs can exert different effects on the endothelium depending on their cell of origin and consequently on their cargo. In physiological conditions, quiescent endothelium secretes EVs that inhibit monocyte activation and suppress endothelial cell activation (Njock et al., 2015), whereas in an inflammatory environment, EVs released by endothelial cells (ECs) can exert angiogenic properties, leading to activation of surrounding ECs (Lombardo et al., 2016). In fact, Lombardo et al. (2016) recently demonstrated that IL-3 enhances EV release by ECs and their pro-angiogenic activity, increasing their miR-126-3p and STAT5 content. Thus, the horizontal transfer of STAT5 into ECs leads to cyclin D1 transcription and tridimensional tube-like structure formation *in vitro*.

In pathological conditions, especially in several inflammatory states, EVs can promote altered angiogenesis. Platelet- and endothelial-derived microparticles were found to be increased, in association with increased thromboxane A2 levels, in patients with septic shock. These EVs displayed a protective effect from hypotension induced by vascular hyporeactivity (Mostefai et al., 2008). Moreover, EVs secreted from renal artery progenitor cells derived from human radical nephrectomy demonstrated to enhance endothelial cell migration when co-cultured with injured endothelial cells. Thus, this study highlighted the feasibility of the use of EVs derived from patient-pro-angiogenic progenitor cells for potential therapeutic autologous cell transplantation for microvasculature endothelial injury (Pang et al., 2017). Furthermore, the role of miRNAs seems to be crucial and van Balkom et al. (2013) showed how the communication in the vascular endothelium is mediated by EVs that stimulate angiogenesis, at least partly, via miR-214. In particular, EC-derived EVs enriched in this miRNA promoted endothelial cell migration and angiogenesis *in vitro* and *in vivo* by preventing cell cycle arrest in recipient ECs (van Balkom et al., 2013). The possibility to exploit this process to rescue senescent cells, with reduced miR-214 levels, by the transfer of EVs derived from miR-214–producing cells provides an EV-application for endothelium-associated diseases.

#### EVs and renal failure

EVs have been involved in the multi-organ dysfunction that typifies sepsis and septic shock (Souza et al., 2015), including AKI. Microvascular injury induced by sepsis induces the release of EVs into the systemic circulation (Ricci and Ronco, 2009), and Zafrani et al. (2012) demonstrated that these EVs have a direct role in the pathogenesis of sepsis and the related AKI, on both coagulation and inflammatory signals, modulating the number of EVs via calpain signaling. In septic patients with AKI, EVs secreted by endothelial progenitors and intravenous injected can reach endothelial and TECs, and have direct effects on cultured hypoxic TECs (Bitzer et al., 2012).

EVs were also reported to contribute to renal failure associated with hemolytic uremic syndrome induced by infection of enterohemorrhagic Escherichia Coli. EVs can provide a way to evade the host immune system and transfer bacterial toxin Shiga toxin (Stx) reaching target organs such us kidney. Using a mouse model infected with *E. coli* Stx, Ståhl et al. (2015) showed that Stx circulates bound to blood cell-derived EVs that reached the kidney and are transferred into glomerular and peritubular capillary endothelial cells. Successively, they pass through the basement membrane and enter in podocytes and TECs. After endocytosis, EVs inhibit protein synthesis and lead to glomerular endothelial cell death (Ståhl et al., 2015).

#### EVs in renal fibrosis

EVs derived by nephron cells can mediate the transfer of proinflammatory or profibrotic signals from tubular epithelial and interstitial cells, such as fibroblasts and infiltrating immune cells, mediating common pathways leading to renal fibrosis (Okada, 2013). In 2013, Borges et al. (2013) showed that injured epithelial cells produced an increased number of EVs with defined genetic information to activate fibroblasts. In fact, TECs-derived EVs subjected to *in vitro* hypoxic damage promoted proliferation, α-smooth muscle actin expression, F-actin expression, and type I collagen production in fibroblasts. Among the complex EV content, the authors highlighted the importance of TGF-β1 mRNA, a profibrotic growth factor (Okada, 2013), suggesting its key role in tissue repair/regenerative responses and activation of fibroblasts (Borges et al., 2013). After cell injury, an incomplete repair response can lead to persistent tubule-interstitial inflammation and tissue hypoxia resulting in AKI (Borges et al., 2013).

Taken together, several studies indicate that EVs are implicated in kidney diseases through multiple effects, depending on their cell of origin and context. Indeed, EVs are involved in physiological processes essential for kidney homeostasis, such as cellular signaling, immune response, ion transport and development. Nevertheless, EVs can be also related to disease progression in cancer or renal failure, as described above. Thus, for instance, EVs can promote altered angiogenesis, inflammation and pro-fibrotic processes. These opposing actions of EVs, both beneficial and harmful, are the consequence of the environment in which EVs are produced, as well as, of the target cell they interact. This opens the perspective to modulate EVs and to exploit their function for therapeutic approaches.

## Therapeutic application of EVs in renal pathology

EVs derived from progenitor and stem cells have been shown to modulate important disease processes displaying regenerative properties that may be applicable in renal pathologies.

### EVs in glomerulonephritis

EVs demonstrated a protective effect in experimental Thy1.1 glomerulonephritis in rats induced by complement-mediated mesangial injury (Cantaluppi et al., 2015). Indeed, after iv injection, EVs derived from endothelial progenitor cells localize within injured glomeruli and inhibit mesangial cell activation, leucocyte infiltration and apoptosis. Moreover, EV treatment decreases proteinuria, increases serum complement hemolytic activity, ameliorates renal function, preserves podocyte marker synaptopodin and endothelial antigen RECA-1. EVs' beneficial effect on mesangial cells was due to the inhibition of anti-Thy1.1 antibody/complement-induced apoptosis and C5b-9/C3 mesangial cell deposition (Cantaluppi et al., 2015).

### Angiogenesis

As mentioned above, EVs can promote angiogenesis by transferring pro-angiogenic factors (Lopatina et al., 2016), such as MMP-2 and MMP-9, which support matrix degradation and new blood vessel formation (Taraboletti et al., 2002; Salamone et al., 2016). EVs derived from proangiogenic progenitors showed to elicit a pro-angiogenic phenotype in quiescent human micro- and macrovascular endothelial cells. Their effect is due to the transfer of mRNAs related to nitric oxide synthase (NOS) and PI3K/AKT signaling pathways that favor the organization of human ECs in canalized vessels when subcutaneously injected in a Matrigel matrix together with EVs in severe combined immunodeficient (SCID) mice (Deregibus et al., 2007). In a murine model of hind limb ischemia, endothelial progenitor cell-derived EVs were shown to improve vascularization and limit ischemic injury (Ranghino et al., 2012).

### Tubular regeneration

Several kidney injuries are characterized by damage of the TECs, partially because of hypoxic injury (Borges et al., 2013). It was shown that EVs can induce AKI recovery by reducing tubular apoptosis (Bruno et al., 2012). Several studies demonstrated the beneficial effects of EVs derived from renal resident and exogenous stem/progenitor cells in repairing kidney damage after toxic or ischemic AKI (Bussolati et al., 2005; Lange et al., 2005; Sagrinati et al., 2006; Herrera et al., 2007; Angelotti et al., 2012; Grange et al., 2014). Indeed, EVs derived from human endothelial progenitor cells prevented the renal functional damage in a rat model of ischemia-reperfusion injury (IRI) (Cantaluppi et al., 2012). IRI is a major cause of AKI in humans and it is associated with tubular cell necrosis and endothelial cell dysfunction and loss (Basile et al., 2012). EVs induce functional and morphologic protection from AKI by reducing leukocyte infiltration and apoptosis, and by enhancing TECs proliferation. Endothelial progenitor cell-derived EVs also protect against progression of chronic kidney disease (CKD) after IRI by inhibiting capillary rarefaction, glomerulosclerosis, and tubule-interstitial fibrosis (Cantaluppi et al., 2012). The therapeutic function of EVs is also mediated by the transfer of miRNAs (Collino et al., 2015). Indeed, the healing and protecting effects of EVs were mainly attributed to the transfer of pro-angiogenic miRNAs (miR-126 and miR-296) to hypoxic resident renal cells, changing their phenotype. Interestingly, reducing the miRNA content in EVs, either by Dicer knock-down in proangiogenic progenitors, or using specific antagomirs together with inactivating RNAs, or by treating vesicles with elevated concentrations of RNases, inactivated the observed EV biological activities (Cantaluppi et al., 2012). In a similar model, using mice with ischemic AKI, Burger et al. (2015) showed that the iv administration of EVs derived from human cord blood endothelial colony forming cells, endothelial precursor cells with a high proliferative capacity and pro-angiogenic potential, attenuated renal injury, inducing an amelioration of plasma creatinine, tubular necrosis, and apoptosis. Notably, these EVs are enriched in miR-486-5p and protect mice against kidney IRI by transferring the miRNA to ECs and targeting the PTEN/Akt pathway. The *in vivo* potent protective effects were associated with decreased PTEN levels in kidney and activation of Akt. These effects were blocked by inhibition of miRNA, suggesting that the transfer of miR-486-5p to ECs plays an important role in preventing apoptosis (Viñas et al., 2016). Notably, a recent work of Ranghino et al. (2017) has demonstrated that EVs derived from a resident population of MSCs within the glomeruli (Gl-MSCs) (da Silva et al., 2006; Bruno et al., 2009) displayed a regenerative effect on AKI induced by IRI in SCID mice. These EVs improve kidney function and reduce the ischemic injury through the activation of TEC proliferation. Moreover, their effect was mediated by the miRNA cargo, including a group of miRNAs whose predicted target genes are involved in various biological processes, such as cell communication, nucleic acid metabolism, regulation of cell growth, gene expression, and transport. These miRNAs may influence the pro-regenerative process triggered by Gl-MSC-EVs (Ranghino et al., 2017). This work confirms previous findings that EVs from kidney-derived MSC are involved in the recovery from AKI following IRI by promoting the proliferation of peri-tubular capillary ECs and decreasing peritubular microvascular rarefaction, possibly by acting as carriers of pro-angiogenic signals (Choi et al., 2014). In addition, several works demonstrated that the therapeutic effect of EVs derived from MSC (MSC-EVs) (Herrera et al., 2007; Bruno et al., 2012; Zhang et al., 2016) was mainly due to the transfer of their miRNA content (Collino et al., 2015) in AKI and other renal injuries. Moreover, EVs derived from other stem cells, such as human liver stem cells (HLSCs), showed to be effective in AKI recovery *in vivo* (Herrera Sanchez et al., 2014).

### Fibrosis

Interestingly, erythropoietin (EPO) showed to protect renal tubular basement membrane through EVs in a mouse model of renal tubule-interstitial fibrosis induced by unilateral ureteral obstruction. This molecule fostered bone marrow cells to release EV-containing miR-144, which was able to inhibit tPA/MMP9-mediated proteolytic network and MMP9 into the mouse kidney (Zhou et al., 2016). Moreover, MSC-EVs have been reported to relieve renal fibrosis (Gatti et al., 2011; Du et al., 2013) and EPO can enhance their effect in protecting the kidney from fibrosis-related damage. Indeed, MSC-EVs incubated with EPO showed a greater benefit in unilateral ureteral obstruction *in vivo* and *in vitro*. The EPO treatment increased miRNA content of EVs in about 70% of cases, probably contributing to an enhanced renal protection from injury (Wang et al., 2015).

### Diabetes

Diabetes is the main driver of CKD in the western world (Ritz and Orth, 1999), and almost 40% of diabetic patients develop diabetic nephropathy (DN), one of the most severe complications in diabetes (Jiang et al., 2016). Early stage DN pathological features include podocyte damage/loss (Forbes and Cooper, 2013), and recent findings suggest that EVs derived from conditioned medium of urine-stem cells may prevent renal injury in diabetes by promoting cell survival and vascular regeneration and by preventing apoptosis of podocytes (Jiang et al., 2016). Indeed, Jiang et al. evaluated the effects of weekly tail intravenous injection of these EVs on kidney injury and angiogenesis in a streptozotocin-induced Sprague–Dawley rat model. They found that EVs reduce the volume of urine and microalbuminuria and avoid apoptosis of podocytes and TECs. Moreover, EVs suppressed the overexpression of caspase-3, and increased proliferation of glomerular endothelial cells. Moreover, *in vitro* analysis revealed that EVs could reduce podocyte apoptosis induced by high glucose. The authors suggested that TGF-β1, angiogenin, and bone morphogenetic protein-7 growth factors carried by EVs may be instrumental of their beneficial effects (Jiang et al., 2016).

DN is also characterized by mesangial cell hypertrophy (Forbes and Cooper, 2013) and both MSC- and HLSC- derived EVs demonstrated to preserve mesangial cells from hyperglycemia-induced collagen production/hyperglycemic damage. This occurs via the horizontal transfer of functional miR-222, resulting in STAT5 down-regulation, and a decrease in miR-21 content, TGFβ expression and matrix protein synthesis (Gallo et al., 2016).

Overall these studies underline the beneficial and protective action of EVs derived from progenitor and stem cells in renal pathological processes, by modulating fibrosis, tubular and glomerular damage, and angiogenesis. Hence, these findings lay the groundwork for therapeutic applications of EVs in nephrology either by elucidating important pathways of kidney recovery or through the evidence of cell-derived EV treatment.

## EVs as renal disease biomarkers

EVs secreted by renal and urologic tract cells convey in urine, bringing important information about the pathophysiological state of the genitourinary system (Musante et al., 2012; Raimondo et al., 2013). Importantly, uEVs can be easily and non-invasively isolated from patients providing a useful starting material for multiple downstream analysis for biomarkers discovery. Several protocols exist for EV isolation (Royo et al., 2016a), including ultracentrifugation, filtration, immune-affinity and microfluidic-based methods, size exclusion chromatography and precipitation (Zhou et al., 2006a; Gámez-Valero et al., 2015; Deregibus et al., 2016). These different methods may vary in purity of the resulting EVs and in the complexity of the protocol, and therefore in their possible clinical application. To avoid the analysis of urinary contaminants, such as shed cells or unbound proteins, some technical precautions are needed including centrifugations or filtration steps, addition of protease inhibitors, and pH control (Zhou et al., 2006a; Zhao et al., 2017). The advantage of EVs as biomarker is the possibility to obtain different sets of information. Indeed, uEV cargo can be analyzed either for its protein content using liquid chromatography, mass spectrometry and enzyme linked immunosorbent assays (ELISA), or for mRNA and miRNA expression through qRT-PCR based methods (van Balkom et al., 2011; Wang et al., 2017). The selected EV analysis is advantageous in respect to the general protein or mRNA investigation in biofluids because it can improve the sensitivity and precision of biomarkers detection. For instance, Skog et al. (2008) identified the tumor EV-carried mRNA of a specific variant of the VEGF-receptor (VEGFvIII), able to predict the therapy response in glioblastoma. Furthemore, EV protein content accounts for about 3% of the total proteins in normal urine (Raimondo et al., 2013; Nawaz et al., 2014), whereby their proteome could better reflect the cellular processes associated with the pathogenesis of genitourinary system as compared with the native urine (Raimondo et al., 2013).

For these reasons, urine represents an ideal fluid for downstream analysis. The study of uEVs can also improve the understanding of the biological mechanisms that occur in cancer or other pathologies and be potentially used for therapies.

### Cancer

In recent years, EVs have gained considerable attention not only as mediators of cancer intercellular signaling but also as potential sources of biomarkers to monitor cancer progression by a non-invasive procedure (Nawaz et al., 2014; Becker et al., 2016). In fact, tumors are characterized by an increase secretion of EVs (De Palma et al., 2016a) that contain a tumor molecular signature (Fais et al., 2016) and flow in biofluids, such as blood and urine. In blood, the amount of serum-EVs, for example, was shown to correlate with a poor prognosis in cancer patients (Mitchell et al., 2008) and their level of vesicular Glypican-1 can provide diagnostic information in early pancreatic cancer (Welton et al., 2016). Similarly, uEVs may help to timely diagnose and monitor genitourinary malignancies (Bryzgunova et al., 2016). Here we will describe the latest findings regarding the use of EVs as biomarkers in genitourinary malignancies, which classically include prostate, kidney, and bladder cancer (Nawaz et al., 2014). A list of candidate uEV-specific cancer markers is summarized in Table 1.

**Table 1:** Candidate uEV biomarkers for urologic malignities.

**Pathology**	**Regulation**	**Marker**	**Source**	**Method**	**References**
**PROSTATE CANCER**					
	↑	α1-antitrypsin, histone H2B1K	Patients	MALDI-TOF spectrometry	Lin et al., 2016
	↑	α1-integrin, β1-integrin	Cell lines and patients with metastatic cancer	LC-MS/MS and Western blot	Bijnsdorp et al., 2013
	↑	PSA, PSMA	Patients	Western blot	Mitchell et al., 2009
	↑	Kininogen-1, afamin, cardiotrophin-1, legumain, FGF19, IGFBP2, IGFBP5, CCL16, CD226	Patients	SEC and SOMAscan™ assay	Welton et al., 2016
	↓	MICA, vWF, A disintegrin, ADAMTS1			
	↑	TMPRSS2:ERG, PCA-3	Patients	Nested PCR	Nilsson et al., 2009
				qRT-PCR	Dijkstra et al., 2014
	↑	BIRC5, ERG, PCA-3, TMPRSS2:ERG, TMPRSS2	Patients	qRT-PCR	Motamedinia et al., 2016
	↓	CDH3	Patients	qRT-PCR	Royo et al., 2016b
	↓	miR-34a	Cell lines and patient gene expression datasets	qRT-PCR and Clinical Datasets	Corcoran et al., 2014
**RENAL CARCINOMA**					
	↑	MMP-9, CP, PODXL, DKK4, CAIX	Patients	LC-MS/MS and Western blot	Raimondo et al., 2013
	↓	AQP1, EMMPRIN, CD10, dipeptidase 1, syntenin-1			
	↓	GSTA1, CEBPA, PCBD1	Patients with ccRCC	Oligonucleotide arrays and qRT-PCR	De Palma et al., 2016b
**BLADDER CANCER**					
	↑	α1-antitrypsin, histone H2B1K	Patients	MALDI-TOF spectrometry	Lin et al., 2016
	↑	Resistin, retinoic acid-induced protein 3, Gs α subunit, EPS8L1, EPS8L2, GTPase NRas, Mucin 4, EDH4	Patients	LC-MS/MS	Smalley et al., 2008
	↓	Galectin-3-binding protein			
	↑	TACSTD2	Patients	LC-MS/MS and ELISA	Chen et al., 2012
	↑	LASS2, GALNT1	Patients	Oligonucleotide array and PCR	Perez et al., 2014
	↓	ARHGEF39, FOXO3			

*Marker: PSA, prostate-specific antigen; PSMA, prostate specific membrane antigen; FGF19, fibroblast growth factor 19; IGFBP2 and IGFBP5, insulin-like growth factor-binding proteins 2 and 5; CCL16, C-C motif chemokine-16; MICA, MHC class I polypeptide-related sequence-A; vWF, von Willebrand factor; ADAMTS1, metalloproteinase with thrombospondin motifs 1; TMPRSS2:ERG, fusion mutation between the androgen driven gene transmembrane protease serine 2 (TMPRSS2) and the oncogene ETS-related gene (ERG); PCA-3, prostate cancer antigen 3; BIRC5, survivin; ERG, ETS-related gene; TMPRSS2, transmembrane protease serine 2; CDH3, Cadherin 3 type 1; MMP-9, matrix metalloproteinase 9; CP, ceruloplasmin; PODXL, podocalyxin; DKK4, dickkopf related protein 4; CAIX, carbonic anhydrase IX; AQP1, aquaporin-1; EMMPRIN, extracellular matrix metalloproteinase inducer; CD10, neprilysin; GSTA1, glutathione S-Transferase Alpha 1; CEBPA, CCAAT/enhancer-binding protein alpha; PCBD1, pterin-4 alpha-carbinolamine dehydratase 1; EPS8L1 and EPS8L2, epidermal growth factor receptor kinase substrate 8 like protein 1 and 2; EDH4, EH domain containing protein 4; TACSTD2, tumor-associated calcium-signal transducer 2; LASS2, ceramide synthase 2; GALNT1, polypeptide N-acetylgalactosaminyltransferase 1; ARHGEF39, Rho guanine nucleotide exchange factor 39; FOXO3, forkhead box O3*.

*Source: ccRCC, clear cell renal cell carcinoma*.

*Method: LC−MS/MS, liquid chromatography-tandem mass spectrometry; SEC, size-exclusion chromatography; qRT-PCR, quantitative real-time PCR*.

#### Prostate cancer

PCa is the most frequent cancer in men (Crawford et al., 2017). Considerable evidences suggest the utility of uEVs for PCa diagnosis, highlighting how their analysis could represent a non-invasive method to evaluate and monitor PCa alterations. Indeed, EVs released by prostate cells can be detected in the urine after their secretion via prostate ejaculatory ducts (Bryzgunova et al., 2016). Several studies investigated the proteomic cargo of prostate-derived uEVs (Pisitkun et al., 2004; Zhou et al., 2006a; Lu et al., 2009; Bijnsdorp et al., 2013). Increased levels of β1-integrin and α1-integrin were detected in uEVs of patients with metastatic PCa, compared with patients with non-metastatic disease or benign prostatic hyperplasia (BPH) (Bijnsdorp et al., 2013). Prostate stem cell antigen and prostate-specific membrane antigen have also been identified in uEVs of patients with PCa (Nyalwidhe et al., 2013; Principe et al., 2013; Drake and Kislinger, 2014). Mitchell et al. (2009) found prostate markers PSA and PSMA in EVs from patients' samples compared to EVs from healthy controls. Welton et al. (2016) demonstrated that it is possible to identify vesicular proteins of blood or urine origin indicative of treatment failure and progressive disease in PCa and discriminate newly diagnosed from progressive PCa. By proteomic analysis techniques, they found proteins with known associations with PCa, including insulin-like growth factor binding proteins and kininogen-1, and identified novel biomarkers elevated during progression, such as Afamin, cardiotrophin-1, legumain, and others (Welton et al., 2016). Moreover, Nilsson and colleagues showed that prostate-related genes could be successfully detected in uEVs and some transcriptomic changes have also been identified, including those affecting the expression of TMPRSS2 and PCA-3 (Nilsson et al., 2009; Dijkstra et al., 2014). Interestingly, a pilot study of Motamedinia et al. (2016) used uEV marker analysis to differentiate patients with biopsy proven PCa from those with negative prostate biopsies with very good accuracy (81%). In particular, the presence in uEVs of prostate specific fusion mutation (TMPRSS2:ERG) between the androgen driven gene transmembrane protease serine 2 (TMPRSS2) and the oncogene Ets Related Gene (ERG) correlated with the gene expression in radical prostatectomy tissue (Motamedinia et al., 2016). Notably, a clinically validated diagnostic test for PCa, based on liquid biopsy, is now available and it allows to discriminate low grade and high grade PCa by measuring uEV expression levels of PCA-3 and ERG (McKiernan et al., 2016). Royo et al. (2016b) showed that uEVs from PCa exhibit different physical and biological properties compared to BPH. The transcriptome analysis revealed decreased abundance of Cadherin 3 type 1 (CDH3) in uEV from PCa patients, reflecting the expression of this cadherin in the prostate tumor and suggesting its tumor suppressive activities in PCa (Royo et al., 2016b).

Other studies analyzed miRNA carried by uEVs. The miRNA profiling in EV derived from PC-3 cancer cells and RWPE-1 normal prostate epithelial cells revealed a panel of 80 miRNAs specifically carried by PCa derived EVs (Hessvik et al., 2012). Corcoran et al. (2014) defined a panel of miRNAs as potentially metastatic biomarker of PCa. According with the work previously described (Hessvik et al., 2012), miR-34a was decreased in PCa and the authors suggested it may be used to discriminate between PCa and BPH. In addition, BCL-2, a well-known anti-apoptotic gene, has been described as target for miR-34a (Corcoran et al., 2014). Finally, PCa-derived EVs showed to play a physiopathological role in cancer progression and development. Babiker et al. (2006) observed that PCa-derived EVs carry protein kinase A that inactivate the complement cascade, thus protecting cancer cells from complement-mediated cell lysis, and CD59 that protect PCa cells from destruction in the microenvironment (Babiker et al., 2005).

#### Renal carcinoma

Renal cell carcinoma (RCC) represents more than 2% of tumors in humans worldwide and renal biopsy is still the gold standard diagnostic procedure, though it is invasive and not suitable for all patients (De Palma et al., 2016b). To date, several studies describe uEVs as promising potential biomarkers. uEVs from RCC patients showed a differential lipid composition, compared to those of healthy control subjects (Del Boccio et al., 2012), and contained RCC-specific protein substantially and reproducibly different from control subjects (Raimondo et al., 2013). Specifically, RCC-uEVs were enriched in MMP-9 and Dickkopf related protein 4, proteins correlated with disease progression and metastatic potential. Moreover, in these vesicles, others proteins were reduced, including AQP1, EMMPRIN, Neprilysin, Dipeptidase 1, and Syntenin-1. The transcriptome content of uEVs from RCC can also be useful to diagnostics and classification. Three transcripts (GSTA1, CEBPA, and PCBD1) are reduced in EVs derived from clear cell renal cell carcinoma patients with respect to healthy subjects and patients with other types of RCC. These alterations are specific and disappear 1 month after partial or radical nephrectomy (De Palma et al., 2016b). However, the information contained in uEVs can represent not only potential biomarkers, but also therapeutic targets. Interestingly, it was shown that a long non-coding RNA (lncRNA) activated in RCC is transferred by EVs and confers sunitinib resistance to sensitive cells by competitively binding miR-34/miR-449, thus promoting AXL and MET expression in RCC cells (Qu et al., 2016).

#### Bladder cancer

Bladder cancer is a frequent malignancy in developed countries, second only to PCa among genitourinary tract malignancies (Nawaz et al., 2014). Several bladder cancer-related-proteins were found in patients' uEVs and could be used as diagnostic or prognostic markers. Components of the epidermal growth factor (EGF) pathway, the α subunit of the G protein Gs, retinoic acid protein 3, and resistin are over-represented in uEVs of patients and potentially involved in tumor progression (Smalley et al., 2008). Tumor-associated calcium-signal transducer 2 (TACSTD2) is highly expressed in uEVs of patients with bladder cancer and was proposed as a biomarker (Chen et al., 2012). The protein EGF-like repeat and discoidin I like domain-containing protein 3 (EDIL 3), an integrin ligand implicated in angiogenesis, is delivered by bladder cancer-derived EVs and might promote cancer progression (Beckham et al., 2014). Moreover, uEVs from bladder-cancer patients showed to carry transcripts for LASS2 and GALNT1, involved in cancer progression and metastasis (Perez et al., 2014), and miR-1224-3p, miR-15b, and miR-135b, which correlate with a positive bladder cancer diagnosis, as well as the ratio miR-126:miR-152 (Huang et al., 2013). Finally, a recent study has shown that uEVs from patients with high-grade bladder cancer express two proteins (α1-antitrypsin and histone H2B1K) whose levels are significantly correlated with disease grades. The proteins are involved in tumor development and are potential uEV biomarkers predicting the risks of recurrence and progression (Lin et al., 2016). Another study observed that EVs from cultured bladder cancer cells incubated with tumor cells activate signaling pathways leading to inhibition of apoptosis, suggesting they may play a role in tumor progression (Yang et al., 2013).

### Non-cancer diseases

uEVs may offer easily accessible markers of kidney injury that reflect tubular and glomerular damage (Ranghino et al., 2015). The analysis of uEVs content (including mRNAs, proteins, and miRNAs) may provide a more precise estimation of the extent of glomerular/tubular damage and possibly also discriminate the type of injury (Miranda et al., 2010; Turco et al., 2016; Ichii et al., 2017). To date, several studies on uEVs have described potential biomarkers associated to kidney injury and Table 2 summarizes the most relevant findings that will be discussed here.

**Table 2:** Summary of candidate uEV biomarkers for renal non-tumoral pathologies.

**Disease**	**Regulation Marker**		**Human/animal model**	**References**
Renal transplantation	↑	NGAL	Patients with DGF after kidney transplantation	Alvarez et al., 2013
Tubular damage	↑	AFT3	Cisplatin-induced AKI and I/R in mice, AKI patients	Zhou et al., 2008
			Sepsis-induced AKI in mice, patients	Panich et al., 2017
			AKI patients	Chen et al., 2014
	↑	fetuin-A	Cisplatin-induced AKI in rats, ICU patients with AKI	Zhou et al., 2006b
	↓	AQP1	I/R in rats, transplant patients	Sonoda et al., 2009
	↑	NHE3	ARF patients	du Cheyron et al., 2003
Glomerular disease	↑	*WT-1*	DM1 patients	Kalani et al., 2013
			CG in mice, FSGS patients	Zhou et al., 2013
	↑	OPG	CKD patients	Benito-Martin et al., 2013
	↓	miR-155, miR-424		
	↓	aminopeptidase N, vasorin precursor	IgAN vs. TBMN patients	Moon et al., 2011
	↑	α-1-antitrypsin, CP		
	↑	miR-26a	LN patients	Ichii et al., 2014
	↑	ADAM10	LN and IgAN patients	Gutwein et al., 2010
Kidney fibrosis	↓	miR-29c	CKD patients	Lv et al., 2013
			LN patients	Sole et al., 2015
	↓	CD2AP	CKD patients	Lv et al., 2014
	↑	E-cadherin, N-cadherin	Patients with PUVs	Trnka et al., 2012
	↓	TGF-β1, L1CAM		
	↓	miR-26a	KD in dogs	Ichii et al., 2017
	↑	miR-21a		
	↓	miR-181a	CKD patients	Khurana et al., 2017
Diabetic disease	↑	miR-320c	Type 2 DN patients	Delić et al., 2016
	↑	miR-15b, miR-34a, miR-636, miR-192	DM2 patients	Eissa et al., 2016
				Jia et al., 2016
	↑	miR-451-5p, miR-16	Streptozotocin-induced DM1 in rats	Mohan et al., 2016
	↑	miR-130a, miR-145	DM1 patients with DN	Barutta et al., 2013
Other diseases	↑	TMEM2	ADPKD patients	Hogan et al., 2015
		S100-A8, annexin A1		Pocsfalvi et al., 2015
	↓	NKCC2	Patients with Bartter syndrome type I	Gonzales et al., 2009
	↓	NCC	Gittelman's syndrome patients	Joo et al., 2007

*Marker: NGAL, neutrophil gelatinase- associated lipocalin; AFT3, activating transcriptional factor 3; AQP1, aquaporin-1; NHE3, Na/H exchanger isoform 3; WT-1, Wilms' tumor 1; OPG, osteoprotegerin; CP, ceruloplasmin; L1CAM, L1 cell adhesion molecule; NKCC2, sodium-potassium-chloride co-transporter protein; TMEM2, transmembrane protein 2; NCC, thiazide-sensitive sodium-chloride (Na-Cl) cotransporter*.

*Model: DGF, delayed graft function; AKI, acute kidney injury; I/R, ischaemia/reperfusion injury; ICU, Intensive Care Unit, ARF, acute renal failure; FSGS, focal segmental glomerulosclerosis; CG, collapsing glomerulopathy; CKD, chronic kidney disease; DM1, type 1 diabetic patients; DM2, type 2 diabetic patients; DN, diabetic nephropathy; IgAN, IgA nephropathy; TBMN, thin basement membrane nephropathy; PUVs, posterior urethral valves; KD, kidney disease; LN, lupus nephritis; ADPKD, autosomal dominant polycystic kidney disease*.

#### Renal transplantation

A recent study by Dimuccio et al. (2014) showed that uEVs expressing the progenitor marker CD133 (CD133+ uEVs) and typical glomerular and proximal tubular markers may represent an indicator of renal functionality. Indeed, CD133+ uEVs are present in the urine of normal subjects, but not of patients with end stage renal disease, possibly reflecting the activity of CD133+ cells correlated to renal repair after injury (Dimuccio et al., 2014). uEVs may also be useful for the evaluation of the allograft damage after renal transplant, since uEVs of patients with delayed graft function are enriched of neutrophil gelatinase-associated lipocalin (NGAL) protein, an emerging biomarker of AKI and delayed graft function (Alvarez et al., 2013). In contrast, the mRNA level of NGAL and other proteins associated to kidney injury (kidney injury molecule-1, cystatin C, and interleukin-18) does not increase after transplant (Peake et al., 2014), highlighting a variability in mRNA packaging in uEVs and the need for further studies. Anyway, in transplanted patients, uEVs may represent a potential source for markers of drug toxicity. For example, a significant increase of NKCC2 and Na-Cl co-transporter was found in uEVs from cyclosporine-treated kidney transplanted patients compared with the controls (Esteva-Font et al., 2014).

#### Tubular damage

To date, some uEV potential biomarkers for tubular damage in AKI have been identified, including activating transcriptional factor 3 (AFT3) (Zhou et al., 2008), fetuin-A (Zhou et al., 2006b), and AQ1 (Sonoda et al., 2009). Zhou et al. (2008) demonstrated that there is a significant increase of AFT3 protein level in uEVs, but not in whole urine, of mice with AKI, highlighting the diagnostic potential of uEVs. This marker remains elevated for 24–48 h and increases before the increase in serum creatinine. Importantly these results were subsequently confirmed in four patients with AKI (Zhou et al., 2008). According to these data, Chen et al. (2014) found a 60-fold increased level of AFT3 mRNA in AKI patients compared with normal controls. Recently, Panich and colleagues (Panich et al., 2017) identified ATF3 protein expression in uEVs as feasible biomarker for sepsis-induced AKI in patients. Another uEV biomarker of AKI is fetuin-A, which increases 52.5-fold after damage and precedes the increase of serum creatinine in both animal models and patients (Zhou et al., 2006b). In contrast, the levels of AQ1 rapidly decreased both in a rat model of ischemia/reperfusion injury and in patients immediately after kidney transplantation. The level of uEV-AQ1 seems to positively correlate with its level of apical membrane expression in renal tubules (Sonoda et al., 2009; Abdeen et al., 2016). Similarly, the uEV-content of another member of aquaporin family, AQP2, has proven useful for detection of gentamicin-induced renal injury (Abdeen et al., 2014) and is a potential strong candidate for water balance disorders (Oshikawa et al., 2016). Finally, increased levels of Na^+^/H^+^ exchanger type 3 in uEVs-derived from patients have been reported in acute tubular necrosis, but not in prerenal azotemia and other causes of acute renal failure, suggesting its diagnostic potential in AKI of acute tubular necrosis origin (du Cheyron et al., 2003).

#### Glomerular diseases

The increase or reduction of a podocyte marker may reflect, respectively, a glomerular injury or the podocyte loss in chronic renal damage. Several works agree to associate the podocyte injury to alterations of Wilms' tumor 1 (WT-1) in animal models as well as in patients affected by chronic glomerular pathologies (Kalani et al., 2013; Zhou et al., 2013). WT-1 levels are increased in uEVs from patients with diabetes mellitus type 1 (DM1) with proteinuria compared with patients without proteinuria and high levels negatively correlate with the renal function (Kalani et al., 2013). The reduction of WT-1 or other markers of podocyte damage in patients' uEVs can show a remission from the disease or a response to therapy (Zhou et al., 2013). Recently, it has been reported that uEVs derived from podocyte are higher in patients with DM1, independently from other biomarkers (albuminuria, nephrin) and they may help to detect glomerular injury in uncomplicated DM1 (Lytvyn et al., 2017). Moreover, EVs released by the tip of glomerular podocyte microvilli and positive for the podocyte marker podocalyxin were increased in patients with nephritic syndrome (Hara et al., 2010).

uEVs obtained from patients with CKD, were found to express lower levels of CD2AP mRNA, another podocyte marker possibly correlated with renal dysfunction, proteinuria levels, and the stage of renal fibrosis (Lv et al., 2014). Another study reported an increase of osteoprotegerin, a decoy receptor of the tumor necrosis factor superfamily pro-apoptotic cytokine, marker of inflammation, in CKD patients (Benito-Martin et al., 2013). Alteration of miRNA cargo were also reported in DM1 patients with or without diabetic nephropathy. uEVs derived from microalbuminuric patients were enriched in miR-130a and miR-145, a glomerular marker of mesangial cells induced by TGF-β1. uEVs were characterized by a decrease content of miR-155 and miR-424, which are expressed on podocytes and negatively modulate the signaling of angiotensin II, TGF-β1, and VEGF (Barutta et al., 2013). In adult and pediatric patients with isolated microscopic hematuria, uEVs were shown to differentiate between early IgA nephropathy and thin basement membrane nephropathy. uEVs differently express four biomarkers: α-1-antitrypsin and ceruloplasmin mark the IgA group, whilst aminopeptidase N and vasorin precursor are enriched in the thin basement membrane of the nephropathy group (Moon et al., 2011).

Several miRNAs were also found to be differentially expressed in lupus nephritis and IgA nephropathy patients in respect to controls. For instance, the expression of miR-26a is decreased in glomeruli of nephropatic patients and conversely increased in their uEVs, suggesting its importance as putative biomarker of glomerular injury (Ichii et al., 2014). Similarly, ADAM10, which is normally expressed in differentiated podocytes, can be found in uEVs of lupus nephritis and IgA nephropathic patients, but not in healthy donor uEVs (Gutwein et al., 2010).

#### Kidney fibrosis

In patients with CKD, renal fibrosis was shown to directly influence the miRNA content of uEVs. Lv et al. (2013) demonstrated a significantly reduction in uEVs of miR-29 and miR-200 family of patients with CKD compared with controls and its correlation with renal function and the degree of tubular-interstitial fibrosis. In another work, they showed a decrease in CD2AP mRNA levels and an increase of synaptodin in uEVs from patients (Lv et al., 2014).

In CKD, regardless the presence or the extent of renal damage, uEVs may help to evaluate the risk of developing renal dysfunction. Trnka et al. (2012) showed their potential use as markers of obstructive nephropathy, a leading cause of CKD in children. uEVs collected by patients with posterior urethral valve contained high levels of E-cadherin and N-cadherin, and reduced levels of TGF-β1 and L1 cell adhesion molecule compared with the controls. Remarkably, the level of the pro-fibrotic factor TGF- β1 in uEVs correlated with the glomerular filtration rate. In a recent study in dogs, also the miRNA content of uEVs was shown to reflect kidney disease status. In this regard, Ichii et al. (2017) found some miRNAs associated with altered renal functions and kidney tissue injuries, with miR-26a and miR-21a that would be strong candidates indicating glomerulus and tubule-interstitium damages, respectively. Moreover, miR-181a appeared to be a potential biomarker in CKD patients, being significantly decreased by about 200-fold compared to healthy controls (Khurana et al., 2017). Changes in the levels of uEV-derived miRNAs have been correlated with lupus nephritis (Perez-Hernandez et al., 2015) and its progression to fibrosis (Sole et al., 2015).

#### Diabetic kidney disease

miRNA alteration in uEVs also seemed to be associated to an early renal impairment in patients with type II diabetes. The altered expression of miR-320c may modulate the TGF-β-signaling pathway via targeting thrombospondin 1 (TSP-1) and represents a putative marker for disease progression (Delić et al., 2016). miR-15b, miR-34a, and miR-636 were shown to be significantly up-regulated in uEVs derived from type 2 diabetes patients. The levels of these miRNAs are positively correlated with physiological parameters (serum creatinine, urinary protein creatinine ratio) and probably contribute in the pathogenesis of kidney disease (Eissa et al., 2016). In type 1 diabetes, instead, was reported an increase in miR-451-5p and miR-16 in uEVs collected from a rat model induced by intraperitoneal injection of streptozotocin. Interestingly, in kidney-tissues, the expression of both these miRNAs appeared protective against diabetes-induced kidney fibrosis (Mohan et al., 2016). Analysis of the expression of 226 miRNAs in uEVs from patients with type 1 diabetes with and without DN showed that 22 miRNAs were differentially expressed and miR-145 and miR-130a were enriched in patients with microalbuminuria. miR-145 was increased also in uEVs and within the glomeruli of animal model of streptozocin-induced DN (Barutta et al., 2013). Recently, miR-192 has been found to be differentially expressed in patients with normo- and micro- albuminuria, allowing the detection of an early stage of DN (Jia et al., 2016). In addition to RNAs and miRNAs, uEVs may carry mitochondrial DNA. The mitochondrial DNA decreased in DN patients (Sharma et al., 2013), indicating the alteration of bioenergy supply in kidney cells, may underline the role of bioenergetics metabolism in renal damage progression (Higgins and Coughlan, 2014).

#### Other pathologies

Polycystic kidney disease (PKD) is an inherited kidney disease very common worldwide. The leading causes are mutations in genes encoding for proteins essential to the functioning of primary cilia, including polycystin1 (PC1), polycystin 2 (PC2), and fibrocystin (Yoder et al., 2002). These and others proteins, including Cystin, ADP ribosylation factor–like 6, plakins and complement, were shown to be expressed in uEV of patients (Pisitkun et al., 2004; Gonzales et al., 2009; Salih et al., 2014). Moreover, uEVs of patients with PKD and healthy controls showed to have different lectin profiles (Gerlach et al., 2013). uEVs of patients with PKD displayed an abnormal expression of cystin and ADP ribosylation factor-like 6 (Hogan et al., 2009). Recently, the same group (Hogan et al., 2015) observed a 2-fold increase of transmembrane protein 2 (TMEM2) in urinary vesicles from patients with PKD1 compared with controls. Interestingly, PC1:TMEM2 and PC2:TMEM2 ratio showed to be inversely correlated with kidney volume, providing a non-invasive and non-imaging tool for the monitoring of kidney volume during the progression of the disease. Moreover, the function of TECs during the progression of autosomal dominant polycystic kidney disease (ADPKD) may be monitored through uEVs. A recent study has reported that about a half out of the total proteins identified in uEVs are differentially expressed among patients and controls. Some proteins, such as cytoskeleton-regulating and Ca^2+^-binding proteins, correlate with the pathogenic state of TECs in ADPKD. Notably, S100-A8 and annexin A1 (two Ca^2+^-binding proteins) decrease after treatment with a vasopressin receptor 2 antagonist (Pocsfalvi et al., 2015).

Moreover, solute transporters, such as NaKCl and NaCl cotransporters, are normally expressed in uEVs and were shown to be absent in uEVs from patients with Bartter syndrome type 1 and Gittelman's syndrome, respectively. This indicates that uEVs analysis may be useful in the diagnosis of these genetic disorders (Joo et al., 2007; Gonzales et al., 2009). Finally, it was reported that the content of uEVs can change in presence of kidney infections, for example in Leptospira-infected rats, uEVs showed a differential expression of 25 proteins that can be used to discriminate infected and healthy controls (RamachandraRao et al., 2015).

## Conclusion

Taken together, the reports summarized in this review point out the increasingly recognized relevance of EVs in renal physiopathology. Over the past years, renal EVs were shown to be involved in cell communication among the nephron and growing evidences suggest their key role in physiological renal processes. In parallel, new findings are supporting their importance in renal regeneration and diseases of the genitourinary system, including cancer, but also inflammatory, genetic diseases, glomerular and tubular damage, and many others. Based on these observations, EVs seem to fulfill several complex functions in kidney pathophysiology and reflect the kidney state of health; though, there is still much to discover. The analysis of EVs collected in urines, carrying disease-specific markers, can help to comprehend the kidney metabolic and pathological mechanisms still poorly understood. Importantly, the EVs conveyed in urine represent a suitable source of biomarkers for the improved diagnosis, prognosis, and clinical monitoring of renal diseases. This could allow a rapid and accurate diagnosis of renal diseases, before a significant damage occurs, without invasive methods. Finally, a methodological consensus for EV isolation and definition could solve the discrepancies concerning EV diagnostic significance currently present in the literature (Bryzgunova et al., 2016).

## Future perspectives

To date, current knowledge opens the way for a potential therapeutic application under different aspects. Firstly, pathology progression could be prevented by blocking EVs directly involved in disease pathogenesis. For instance, the mechanism of EV secretion can be targeted to inhibit cancer progression by blocking regulatory proteins, such as Rab GTPases, Rab27a, and Rab27b in tumor cells (Ostrowski et al., 2010). Moreover, as cancer cells could selectively package and secrete doxorubicin within EVs resulting in drug resistance (Shedden et al., 2003), EV blockade could result in an improvement of pharmacological effects.

A second approach is to use renal EVs exhibiting protective effect as a targeted therapeutic tool. In particular, Chen et al. (2014) found that the intra-renal administration of EVs derived from proximal tubular cells and enriched in ATF3 RNA exhibited a protective effect in a mouse model of I/R-induced acute renal injury. This effect could be explained by the inhibition of the secretion of MCP-1, a potent chemokine involved in acute inflammatory and immune reactions, by renal epithelial cells (Chen et al., 2014). In this setting, stem and progenitor cell-derived EVs could also be of benefit for their regenerative properties (Bruno et al., 2016).

EVs could also have a beneficial effect in the treatment of genetic diseases by transferring wild-type molecules to defective cells. For example, MSC-EVs carrying wild-type cystinosin (protein and mRNA) showed to reduce the accumulation of cystine *in vitro* in proximal tubular cells isolated from cystinosis patients (Iglesias et al., 2012).

A final possible therapeutic application comprehends the use of EVs as a drug carrier. Drugs, proteins or RNAs can be loaded into EVs with several methods, such as electroporation, coincubation, transfection, and delivered to recipient cells for cancer and regenerative therapy (Barile and Vassalli, 2017; Luan et al., 2017). For instance, EVs loaded with a siRNA against RAD51 by electroporation were showed to induce a significant reduction of RAD51 transcript in HEK293 and HCT116 colon cancer cell lines upon incubation (Shtam et al., 2013). HEK293- and MSC-derived EVs loaded with a siRNA for PLK-1 could entry into bladder cancer cells *in vitro* and silence the specific gene transcription (Greco et al., 2016). Moreover, MSC-EVs enriched for miR-let7c selectively targeted the fibrotic kidney in an *in vivo* model of unilateral ureteral obstruction and downregulated several pro-fibrotic genes (Wang et al., 2016).

Altogether, these studies underline the multifaceted applications of the EVs and support increasing interest for their full understanding.

## Author contributions

All authors listed have made substantial, direct and intellectual contribution in writing the paper.

### Conflict of interest statement

BB and GC are named as inventors in EV-related patents. The other authors declare that the research was conducted in the absence of any commercial or financial relationships that could be construed as a potential conflict of interest.
